# Evaluation of post-ablation mpMRI as a predictor of residual prostate cancer after focal high intensity focused ultrasound (HIFU) ablation

**DOI:** 10.1016/j.urolonc.2022.07.017

**Published:** 2022-09-02

**Authors:** Yash S. Khandwala, Shravan Morisetty, Pejman Ghanouni, Richard E. Fan, Simon John Christoph Soerensen, Mirabela Rusu, Geoffrey A. Sonn

**Affiliations:** a Department of Urology, Stanford University Medical Center, Stanford, CA; b Department of Radiology, Stanford University Medical Center, Stanford, CA

**Keywords:** Prostate cancer, Magnetic resonance imaging, Focal therapy, High intensity focused ultrasound

## Abstract

**Purpose:**

To evaluate the performance of multiparametric magnetic resonance imaging (mpMRI) and PSA testing in follow-up after high intensity focused ultrasound (HIFU) focal therapy for localized prostate cancer.

**Methods:**

A total of 73 men with localized prostate cancer were prospectively enrolled and underwent focal HIFU followed by per-protocol PSA and mpMRI with systematic plus targeted biopsies at 12 months after treatment. We evaluated the association between post-treatment mpMRI and PSA with disease persistence on the post-ablation biopsy. We also assessed post-treatment functional and oncological outcomes.

**Results:**

Median age was 69 years (Interquartile Range (IQR): 66–74) and median PSA was 6.9 ng/dL (IQR: 5.3–9.9). Of 19 men with persistent GG ≥ 2 disease, 58% (11 men) had no visible lesions on MRI. In the 14 men with PIRADS 4 or 5 lesions, 7 (50%) had either no cancer or GG 1 cancer at biopsy. Men with false negative mpMRI findings had higher PSA density (0.16 vs. 0.07 ng/mL^2^, *P* = 0.01). No change occurred in the mean Sexual Health Inventory for Men (SHIM) survey scores (17.0 at baseline vs. 17.7 post-treatment, *P* = 0.75) or International Prostate Symptom Score (IPSS) (8.1 at baseline vs. 7.7 at 24 months, *P* = 0.81) after treatment.

**Conclusions:**

Persistent GG ≥ 2 cancer may occur after focal HIFU. mpMRI alone without confirmatory biopsy may be insufficient to rule out residual cancer, especially in patients with higher PSA density. Our study also validates previously published studies demonstrating preservation of urinary and sexual function after HIFU treatment.

## 1. Introduction

The current standard treatment modalities offered for localized prostate cancer are effective but associated with well-documented morbidity [1,2]. Radical whole-gland therapy subjects patients with low volume, intermediate risk cancer to potentially significant urinary, sexual, and rectal side effects that may outweigh the oncologic benefits of the treatment [3]. Focal therapy is increasingly utilized as a tissue-preserving modality providing a subset of men with an intermediate option between active surveillance and whole-gland therapy with the intention of reducing side effects while maintaining oncologic benefit [4].

Over the last decade, promising short to medium-term data–up to 5 years–have corroborated the effectiveness of High Intensity Focused Ultrasound (HIFU) focal therapy with overall survival >99% at 5 years and significantly reduced erectile dysfunction and urinary incontinence compared to radical therapy [5,6]. However, over one-third of cases may have residual or recurrent disease after treatment, and follow-up protocols lack standardization [7]. While previously reported failure-free survival rates are high, the lack of routine re-biopsy after ablation in these cohorts makes it uncertain if this success is due to cancer destruction or lack of detection [5,8]. Despite several large studies evaluating HIFU outcomes using the PIRADS system as an indicator for when to biopsy, mpMRI remains unvalidated in the post-ablation setting [6,9,10]. There remains a need to assess post-ablation diagnostic testing by correlating it with whole-gland prostate biopsy histopathology after HIFU therapy.

To address these unmet needs, the primary aim of our study was to evaluate the ability of post-ablation MRI and PSA testing to predict residual cancer after HIFU treatment in a prospectively enrolled cohort of men with localized prostate cancer who underwent per-protocol repeat biopsy. We also sought to validate functional and oncologic outcomes of focal HIFU therapy in the same cohort.

## 2. Patients and methods

### 2.1. Study design and patients

**Fig. 1.**
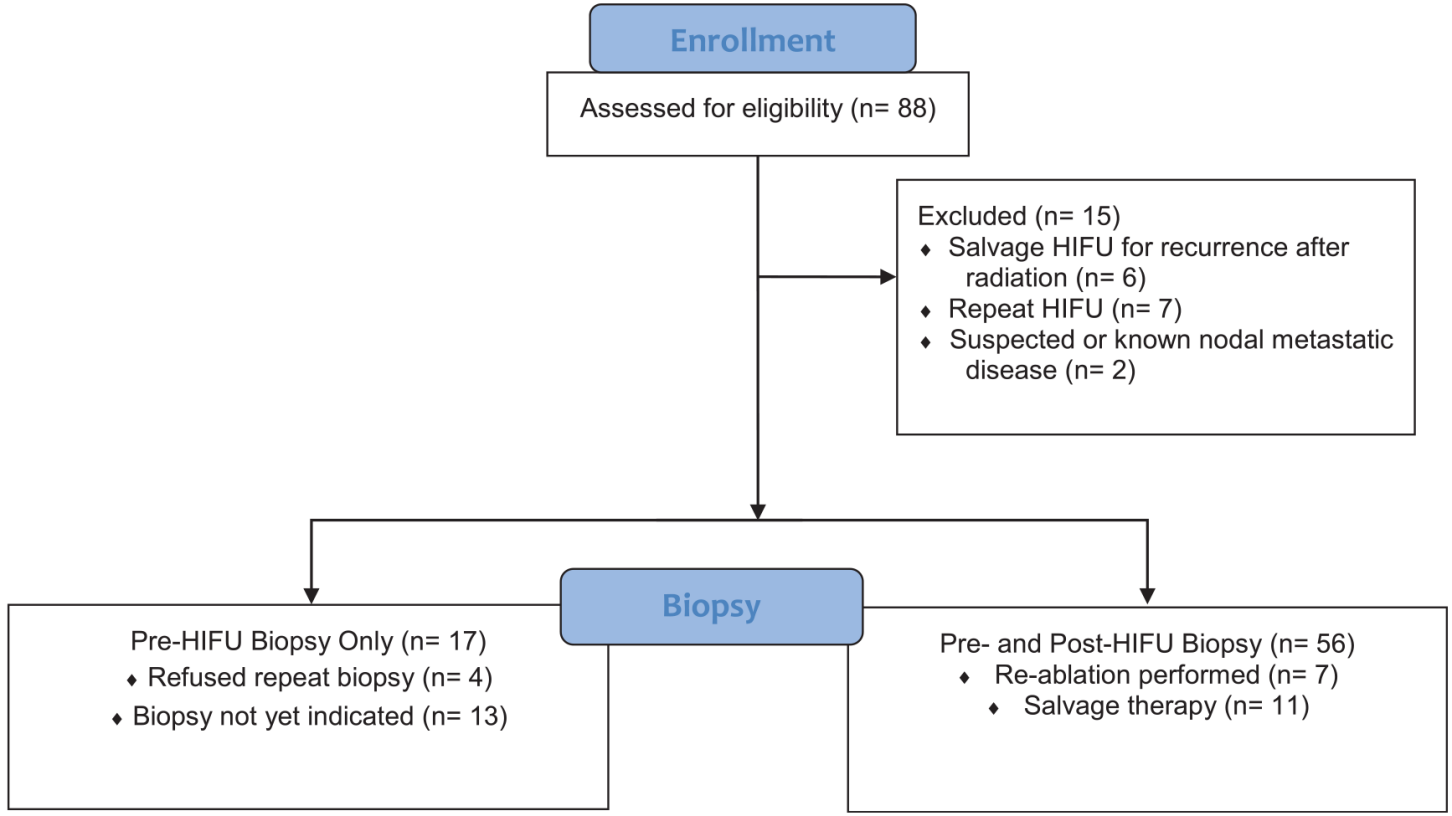
CONSORT flow diagram of patients included in the final analysis.

We prospectively enrolled 88 consecutive men who underwent HIFU focal ablation using a Sonablate 500 device (Sonablate Corp., Charlotte, NC) by a single urologist in a single-armed study at Stanford (IRB# 52438) between May 2016 and May 2021. Six men who underwent salvage HIFU after prior radiation therapy were excluded and 7 men had repeat HIFU so only their first treatment was included in the study. Men with suspicious or known metastatic nodal disease at the time of therapy (n = 2) were excluded from the oncological analysis though were included in the functional outcomes assessment. Fig. 1 displays a CONSORT flow diagram of included patients. Prior to treatment, informed consent was obtained for inclusion into the data registry.

Each subject underwent multiparametric MRI (mpMRI) including T2, diffusion weighted imaging and dynamic post-contrast imaging at 3-Tesla for disease localization and a ≥ 12-core systematic and in most cases fusion-targeted prostate biopsy. Not all pre-ablation MRIs were performed at Stanford. Thirty-five men (48%) underwent biopsy at our institution, while 38 (52%) had a biopsy at an outside facility. Sixty-two men (85%) had the MRI performed prior to biopsy, while the remainder underwent MRI following systematic biopsy. Pre-ablation mpMRI was reviewed by a urologist often with the input of a genitourinary specialized radiologist. All outside biopsy pathology was re-reviewed at our institution prior to treatment. Focal HIFU treatment was offered primarily to patients with intermediate risk localized prostate cancer who had unilateral and unifocal tumors visible on imaging (Table 1). However, patients with suboptimal disease characteristics who sought HIFU treatment outside of this selection criteria were still offered enrollment into the study. All patients were stratified using D’Amico risk groups [11].

Functional outcomes were obtained from electronic surveys distributed using REDCap (Nashville, Tennessee). Pre-treatment surveys were administered 3 days before treatment and then at 3-month intervals for the first year followed by 6 month intervals for ensuing years. The validated surveys included Sexual Health Inventory for Men (SHIM), International Prostate Symptom Score (IPSS), and the Expanded Prostate Cancer Index Composite (EPIC-26) questionnaires, though the latter contained significant missing data and was omitted [2,12,13]. Other missing data was supplemented through chart review by the authors. Minimum follow-up was for 6 months.

PSA tests were ordered at baseline and then at 3 month intervals for the first year and 6 month intervals for every subsequent year. The follow up protocol also included a prostate mpMRI and MRI-US fusion biopsy at 12 months after treatment. Biopsy included 12-core systematic sampling, 2-cores targeting the treated area, and additional targeted sampling of any lesions identified on MRI. Four patients declined the recommended follow-up biopsy, and 13 patients were scheduled to undergo biopsy after the completion of this study. PSA values, imaging findings, patient characteristics, and tumor characteristics were extracted from the electronic medical record through chart review. Tumor location, multifocality, and the presence of contralateral disease were obtained through review of radiology and pathology reports.

### 2.2. Description of the intervention

HIFU treatment was performed by a single experienced urologist (GS) using the Sonablate 500 device (Sonablate Corp; Charlotte, NC). The procedures were performed under general anesthesia with the patient in low lithotomy position. MRI-US fusion was provided by PROFUSE software (Grass Valley, CA) which was utilized to import a treatment plan that incorporated mpMRI imaging and targeted and systematic biopsy results. Treatment plans included the MRI-visible tumor(s) and an additional 8-10 mm margin [14]. Tissue change was measured in real-time using a built-in ultrasound backscatter monitoring device that is included in the Sonablate 500 probe. Visual cues, such as cavitation on real-time transrectal ultrasound, were also used by the surgeon to assess the amount of tissue change induced by ablation [15]. Zones of the prostate with inadequate visual or measured change were re-ablated. A 16 Fr. Foley catheter was placed at the end of the case with planned removal in 4 days.

### 2.3. Outcomes

The primary outcome of the study was the evaluation of post-ablation mpMRI and PSA as predictors of persistent clinically significant cancer (Gleason grade group ≥2) on per-protocol post-ablation biopsy. All follow-up mpMRIs were carefully scored by a genitourinary trained radiologist after reviewing the T2, DWI, and DCE sequences (Supplementary Table 1). Diffusion weighted imaging sequences utilized b-values of 0, 50, 800, and 1400 sec/mm^2^. MRI results were obtained through retrospective chart review of imaging reports and were categorized using PI-RADS score groupings. A positive mpMRI was defined as PI-RADS score greater than or equal to 3. We also report our overall functional and oncological outcomes up to 24 months as secondary outcomes. These include erectile function, urinary function and Gleason scores after HIFU. Overall effect on sexual function before and after treatment was measured by the SHIM score; satisfactory sexual function was defined as SHIM ≥21, indicating mild to no erectile dysfunction (ED) [1]. New onset ED was defined as a score of < 3 on the second question of the SHIM survey in men who reported a score of ≥3 at baseline [4,16]. Urinary function was compared using IPSS scores; quality-of-life responses were grouped into 3 categories: pleased, mixed, and displeased.

### 2.4. Statistical analysis

Patient and tumor characteristics were presented using descriptive statistics. Categorical variables were presented as numbers and percentages while continuous variables were presented as means (standard deviation) or medians (interquartile range [IQR]) depending on the distribution of data. Mean SHIM and IPSS scores (including the quality-of-life index) were compared to baseline. Comparisons were performed using the Student’s t-test with a significance level defined as *P* < 0.05. Box and whisker plots were presented to depict the distribution of IPSS and SHIM scores and PSA dynamics.

Biopsy and imaging results before and after treatment were compared as categorical variables using the Chi-square test of independence. A multivariate logistic regression model including the 56 men with follow-up biopsy data was developed to identify peri-operative factors predicting treatment success. All data available to physicians prior to the 12 month biopsy were included with the goal of simulating the decision of whether biopsy is warranted at this time point. Factors accounted for within the models were age, pre-operative Gleason score, number of positive cores, post-ablation prostate volume, 12 month PSA, and PIRADS score on follow-up mpMRI. All statistical analyses were performed using *Stata Statistical Software (*College Station, TX).

## 3. Results

### 3.1. Patient cohort characteristics prior to treatment

Table 1 displays baseline patient and tumor characteristics of all 73 men who met the inclusion criteria and underwent focal HIFU between 2016 and 2021. Median patient age was 69 years [Interquartile range: 66–74 years] and median PSA was 6.9 ng/dL [IQR: 5.3–9.9 ng/dL]. Fifty-eight men (77%) had grade group (GG) 2 or 3 cancer on pre-treatment biopsy and 59 men (79%) were D’Amico intermediate risk.

### 3.2. Predictors of negative post-ablation biopsy

Post-treatment mpMRI PIRADS score was poorly associated with biopsy pathology. Of the 33 men who underwent a repeat biopsy and had a negative MRI, 11 (34.4%) had clinically significant cancer (GG ≥2) on biopsy which defined treatment failure. In contrast, of the 23 men with a positive MRI (PIRADS ≥3), only 8 (35%) had clinically significant cancer (Table 2A).

In a subgroup analysis evaluating only men with negative mpMRI after HIFU focal therapy, patients with a false negative mpMRI (any GG ≥2 cancer detected on combined targeted and systematic biopsy) had a higher PSA density (0.16 vs. 0.07 ng /ml^2^, *P* = 0.01) and mean number of positive biopsy cores (2.6 vs. 0.9, *P* = 0.002) compared to patients with benign or GG1 histology on repeat biopsy. PSA density, calculated using prostate MRI volume and 12 month PSA, above the 75th percentile (>0.10 ng/mL^2^) predicted a false negative mpMRI in 67% of cases (8 men) (Table 2B). The 12-month PSA difference (3.4 vs. 2.3 ng/dL, *P* = 0.11) was not statistically significant.

A 1 ng/dL rise in 12 month PSA compared to pre-treatment baseline was associated with 83% higher odds of failed treatment (OR: 1.83, CI: 1.2–2.9, *P* = 0.01). Besides pre-operative Gleason score (OR: 21.3, CI: 1.2–556, *P* = 0.03) and 12 month PSA, no other clinical variables including age, post-ablation prostate volume, positive cores or PIRADS score were linked with GG ≥2 cancer recurrence after HIFU treatment within a logistic regression model.

### 3.3. Oncologic outcomes

Median PSA after focal HIFU decreased from 6.9 [IQR: 5.3–9.8] to 3.2 ng/dL [IQR: 2.1–4.0] at 24 months. Fig. 3 displays PSA dynamics at baseline, 3 months, 12 months, and 24 months. Prior to treatment, 91% (51 of 56 men) harbored PIRADS 4 or 5 lesions on mpMRI. This declined to 25% (14 of 56 men) after treatment (Table 3).

Of those who underwent post-ablation biopsy, the proportion of men with GG ≥2 cancer on combined targeted and systematic biopsy decreased from 91% (51 of 56 men) at baseline to 34% (19 of 56 men) at 12 months after treatment. When residual GG ≥2 cancer was present, it was nearly always on the ipsilateral side of treatment (17 of 19 men). Seven of the 56 men (13%) had GG ≥2 recurrence within the treatment zone itself. Of the 19 men who had any residual GG ≥2 prostate cancer, 7 elected for repeat HIFU, 7 underwent salvage whole gland treatment with either radiation or radical prostatectomy, and the remaining men are in an active surveillance protocol. Of the 22 men with residual Gleason grade group 1 disease, 11 (50%) had known contralateral GG1 disease from pre-treatment biopsy that was intentionally not treated.

### 3.4. Functional outcomes

Overall, self-reported sexual function of men undergoing treatment remained stable. SHIM scores greater than 20 were reported by 33 of 65 men (51%) at baseline and 11 of 21 men (52%) at 24 months (Table 4). Mean SHIM survey scores 2 years after the procedure did not differ compared to baseline (17.7 vs. 17.0, *P* = 0.75). Of the 40 men who filled out a follow-up survey and had good erectile function prior to treatment (score ≥3 on question #2 (Q2) of the SHIM survey), 33 (83%) maintained that level of function after treatment (Table 4B). Seven men (21%) with satisfactory erections post treatment required a first-time prescription of a phosphodiesterase type 5 inhibitor.

**Fig. 2.**
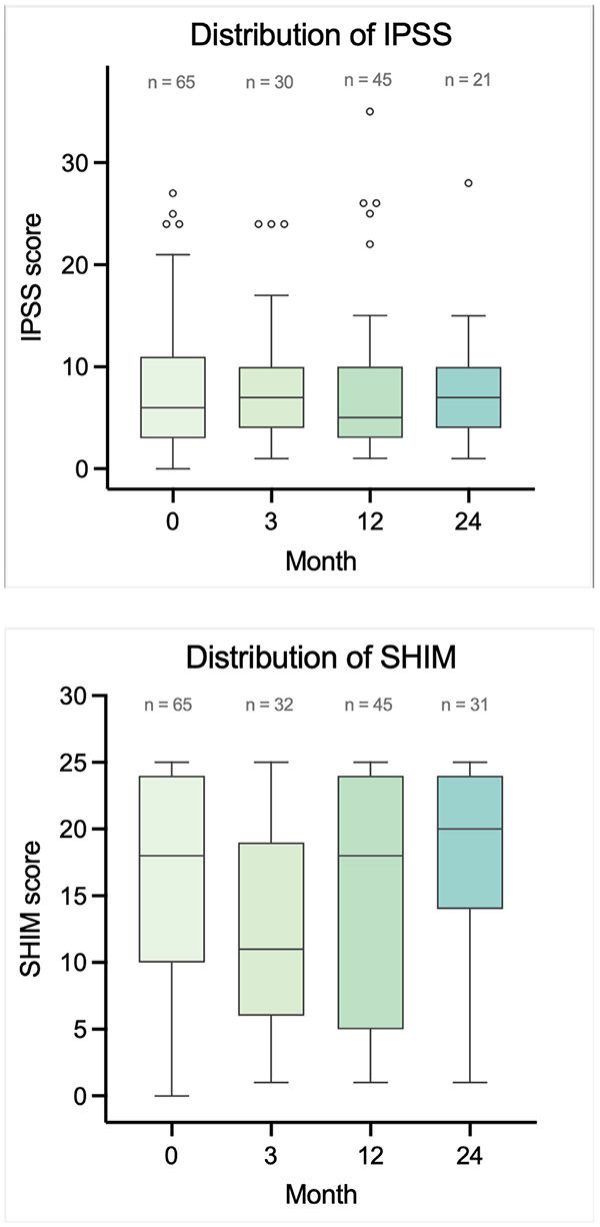
Box and Whisker Plots of the Distribution of SHIM and IPSS Scores at Baseline, 3 Months, 12 Months, and 24 months.

**Fig. 3.**
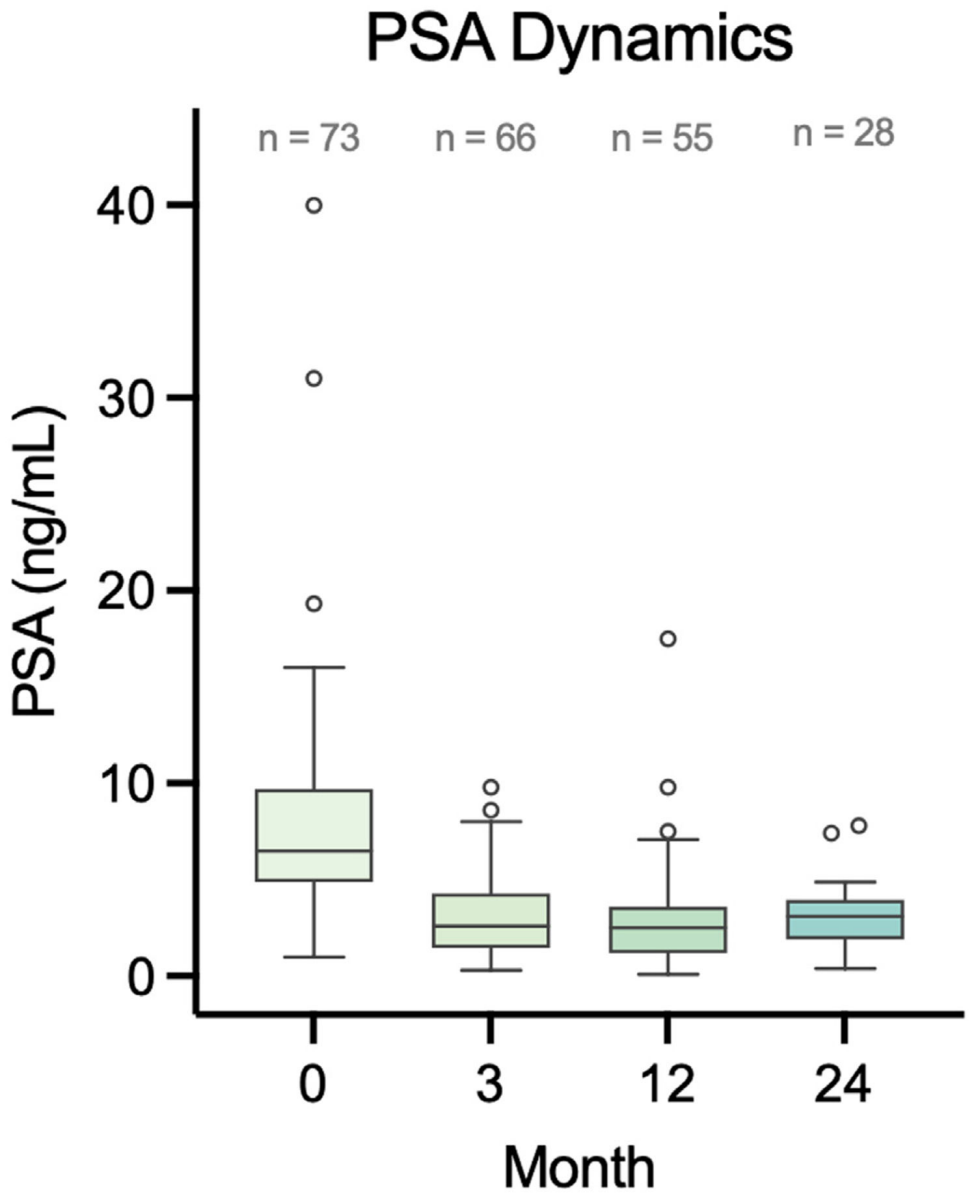
Distribution of PSA Dynamics over Time.

There was no difference in urinary quality of life (QOL) as assessed by the IPSS questionnaire. Mean IPSS score was 8.1 at baseline compared to 7.7 at 24 months (*P* = 0.81). Fig. 2 displays the distribution of IPSS and SHIM scores over time

## 4. Discussion

Our study has 3 key findings. First, the absence of a visible lesion on prostate mpMRI after HIFU therapy did not reliably rule out persistent Gleason grade group ≥2 cancer (GG ≥2) on follow-up biopsy. In fact, 34% of men with a negative post-HIFU mpMRI had GG ≥2 cancer on biopsy. PSA density at 12 months after HIFU, however, did predict false negative mpMRI findings and was independently associated with tumor persistence after HIFU. Only a few prior studies have evaluated the ability of MRI to detect recurrence after focal HIFU. Dickinson et al. reported that mpMRI at 6 to 12 months after HIFU had a low positive predictive value for tumor persistence (14%–44%) [17]. In the same study, MRI only diagnosed 64% to 71% of Gleason pattern 4 disease, suggesting a suboptimal negative predictive value as well. This study was limited by the inclusion of only histopathology from the treated region, and thus true sensitivity was likely even lower. Rouviere et al. also published a small retrospective study of 21 patients that found that contrast-enhanced MRI was unable to predict histological results, though this study was completed over 2 decades ago and relied on older imaging technology [9]. In our study, mpMRI only detected 41% of GG ≥2 cancers found on follow-up systematic and targeted biopsy. Indeed, PIRADS scoring after HIFU ablation remains unvalidated, and clinicians should be careful relying on imaging solely when evaluating oncological outcomes in this setting.

Ideally, biochemical and imaging tools would serve as accurate predictors of treatment outcomes at the whole-gland level to minimize the need for invasive post-treatment prostate biopsy. Therefore, there remains a need to optimize post-ablation imaging and to define other effective post-operative metrics that might better predict HIFU treatment success. Several reasons explain why MRI after HIFU may be unreliable. Hyperthermic modalities induce tissue distortion and a T2 hypointense region within the prostate that can have a similar appearance to cancer. Enhancement on contrast-enhanced T1 phase also occurs due to inflammation and edema after HIFU which can mimic residual cancer in the treated zone [9,18,19]. These issues make it difficult to rely on MRI alone, and especially PIRADS scoring, to assess for tumor persistence.

A second key finding of our study is that focal therapy dramatically reduced the number of men with GG2 or higher cancer. HIFU reduced the proportion of men with GG2 or higher disease from 91% pre-treatment to 34% post-treatment. When present, persistent cancer was most often ipsilateral to the treated area. Our results are similar to previously published reports of a 41% rate of persistent clinically significant disease reported by a single center prospective study evaluating 75 men utilizing stricter inclusion criteria [20,21]. A study by Shoji et al. reported that 20% of 90 men had residual clinically significant cancer or biochemical recurrence [22]. In another study of 1,036 patients with longer follow up, clinically significant cancer after HIFU was found in 16% at 24 months and 36% at 60 months, though routine biopsy was not performed [23]. In a large multicenter prospective trial within the UK, the failure rate was 8% at 3 years, though follow-up included PSA and mpMRI without routine biopsies. This likely under-represented the true rate of recurrence as studies that perform post-treatment biopsy on only a subset of post-ablation cases may miss a substantial number of men with persistent grade group ≥2 cancer [6]. The clinical significance of these missed cancers, which are often small volume, remain unanswered. Other studies use rates of salvage therapy after HIFU to define success [6,24]. In our cohort, 7 men (9%) required salvage surgery or radiation due to ipsilateral persistent clinically significant cancer; this is similar to previously reported rates between 8% and 15% at 36 months [3,6,24].

Importantly, our study utilizes a protocol calling for systematic and targeted biopsies after mpMRI for all patients at 1 year after focal HIFU. This allows for more definitive determination of persistent cancer than that provided when using MRI alone. We found persistent GG ≥2 cancer in 34% of men after treatment though only 13% in-field persistence. Naturally, the follow-up biopsy had a higher density of cores per prostate volume thus some cases of residual disease could be due to prior under-detection. Further reduction of residual cancer rates may require changes in patient selection, treatment planning, and intraoperative ablation technique.

A third important finding of our study is that sexual and urinary function remained relatively unchanged after focal HIFU therapy. Historically, 14% to 48% of men have been reported to develop erectile dysfunction (ED) after focal or hemi-ablation HIFU. This is similar to the 22% of men in our cohort reporting new onset ED (score of <3 on Question 2 of the SHIM survey after being potent at baseline) [24-28]. Some studies used a more lenient cut-off (0 or 1 on question 2) to define erectile dysfunction, but most used the overall SHIM score to identify new cases of ED [26,27]. IPSS scores in our study were also not different after treatment. This is consistent with other HIFU studies that report a <1 point change from mean baseline IPSS up to 12 months after treatment [25,29,30].

Real-world outcomes data of men with prostate cancer undergoing focal HIFU such as ours are important to validate outcomes data in different patient populations [21,23,26]. Men request to undergo focal HIFU treatment for various reasons, sometimes outside of the recommended standard of care. Our study confirms that even without strict inclusion criteria, oncologic outcomes are similar to those previously reported while urinary and sexual function remain minimally affected after focal ablation [6,31,32].

Our study had 5 noteworthy limitations. First, there is a substantial amount of missing data, particularly quality of life surveys, which introduces the possibility of response bias. Despite efforts to efficiently collect data using automated e-survey delivery at set time points before and after treatment, the lack of a dedicated clinical coordinator limited our ability to quickly identify and collect missing data in real-time. There was also variation in pretreatment disease staging and localization which may impact post-treatment outcomes. Second, follow-up is short (median of 13 months), though our primary endpoint focuses on the association of imaging and biopsy results at 1 year [33]. Third, despite recommending targeted biopsy in all patients, not everyone in our study received a follow-up MRI or biopsy. Some of these were due to patient refusal (n = 4). Others were yet to reach the 12 month timepoint after HIFU (n = 13). Still, all patients were asked to undergo 12 month biopsy, and most of our patients did undergo per-protocol biopsy (77%) compared to 36% to 41% in other prominent studies [6,23]. Fourth, our single-center cohort presents a relatively small sample size which may impact the statistical significance of some of the comparisons and limit the generalizability of our findings. Finally, we performed retrospective chart-review and analysis without pre-specified outcomes even though we recruited patients prospectively. This includes the retrospective review of MRI reports. Notably, post-ablation MRI scans were not re-analyzed after the MR-guided fusion biopsy, though all initial reports were completed by experienced radiologists with understanding of expected post-ablation MR changes. We attempted to mitigate the effects of our retrospective analysis by following previously published methods.

In spite of these limitations, our study uniquely identifies the poor negative predictive ability of mpMRI in the post-HIFU setting and presents clinically relevant functional and oncological outcomes by analyzing a population that had mandated targeted and systematic biopsy within 12 months of treatment.

## 5. Conclusion

Focal therapy using HIFU is an emerging treatment modality for the treatment of clinically localized prostate cancer and has shown promise for the eradication of GG2 or higher cancer while minimizing urinary and sexual side effects. However, nearly 30% of patients may still have residual disease after treatment necessitating close followup. Unfortunately, mpMRI is prone to missing positive lesions after focal HIFU therapy, especially in patients with fewer than 2 positive cores and high PSA density. Routine biopsy should be considered for most men without low PSA density after HIFU, though further investigation may reveal a combination of imaging and biomarker testing that may spare some men from repeat biopsy.

## Supplementary Material

Supplementary Material

## Figures and Tables

**Table 1:** Baseline patient and tumor characteristics of cohort.

Total number of men	73
Median age [range]	68.8 [65.5, 73.8]
Mean prostate volume, cc, (median) [range]	45.9 (46) [36, 52]
Mean PSA, ng/mL, (median) [range]	8.1 (6.9) [5.3, 9.9]
PSA Groups, n (%)	
< 10 ng/mL	57 (78.1%)
10-20 ng/mL	14 (19.2%)
> 20 ng/mL	2 (2.7%)
Gleason score, n (%)	
3 + 3 = 6	8 (11.0%)
3 + 4 = 7	35 (48.0%)
4 + 3 = 7	23 (31.5%)
>/ 8	7 (9.5%)
Stage, n (%)	
T1	49 (67.1%)
T2	23 (31.5%)
T3	1 (1.4%)
D’Amico risk group, n (%)	
Low	10 (13.7%)
Intermediate	58 (79.5%)
High	5 (6.8%)
Year of ablation	
2016	1 (1.4%)
2017	3 (4.1%)
2018	4 (5.5%)
2019	27 (37.0%)
2020	25 (34.3%)
2021	13 (17.8%)
Pre-op MRI PI-RADS Score, n (%)	
0	3 (4.1%)
3	3 (4.1%)
4	41 (56.2%)
5	26 (35.6%)

**Table 2:** (A) Association between post-ablation MRI findings and oncological outcomes.

	Post-operative MRI Results			
	Negative MRI	Positive MRI		
		PIRADS 3	PIRADS 4	PIRADS 5
Number of Men with Follow-up Biopsy, n	33	9	11	3
Positive Biopsy, n (%)	21 (63.6%)	8 (88.9%)	9 (81.8%)	3 (100%)
Clinically Significant Positive Biopsy, n (%)	11 (34.4%)	1 (11.1%)	4 (36.4%)	3 (100%)
# of Positive Cores, median [IQR]	1 [0, 2]	2 [1, 3]	1 [0, 3]	7 [4, 10]
PSA Decrease from Baseline (ng/mL), mean (SD)	5.4 (6.4)	5.1 (2.9)	2.7 (1.6)	9.1 (5.8)
	Negative Post-ablation MRI			
Mean (SD)	GG 0 or 1	GG ≥2		
3 Month PSA (ng/mL)	2.7 (2.1)	3.3 (1.5)		*P* = 0.37
12 Month PSA (ng/mL)	2.3 (1.6)	3.4 (1.9)		*P* =0.11
Post-ablation PSA Density (ng/m^2^)	0.07 (.04)	0.16 (.09)		*P* = 0.01

(B) Subgroup analysis of men with negative post-ablation MRI.

**Table 3:** (A) Prostate biopsy (systematic + targeted) Gleason scores before and after HIFU focal ablation. Only patients who underwent post-treatment biopsy (n = 53) were included in this analysis. Chi-Square analysis of distribution of Gleason scores at baseline versus 12 months, *P* = 0.03.

	Baseline Biopsy	Follow-up Biopsy	Follow-up Biopsy Treatment Area
3 + 3 = 6	5 (8.9%)	22 (39.3%)	9 (16.1%)
3 + 4 = 7	27 (48.2%)	9 (16.1%)	1 (1.8%)
4 + 3 = 7	17 (30.4%)	5 (8.9%)	5 (8.9%)
>/ 8	7 (12.5%)	5 (8.9%)	1 (1.8%)
No cancer present	0 (0.0%)	15 (26.8%)	40 (71.4%)
MRI PIRADS Score, n (%)		Baseline MRI	Follow-up MRI
3		2 (3.6%)	9 (16.1%)
4		34 (60.7%)	11 (19.6%)
5		17 (30.4%)	3 (5.4%)
No lesion present		3 (5.4%)	33 (58.9%)

(B) MRI PIRADS score before and after HIFU focal ablation. Only patients who underwent post-treatment mpMRI (n = 59) were included in this analysis. Chi-Square analysis of distribution of PIRADS scores at baseline versus 12 months, *P* = 0.29.

**Table 4a:** Sexual and Urinary Function Scores as measured by IPSS and SHIM Surveys at Baseline, 3 months, 12 months, and 24 months. n (%). IPSS =International Prostate Symptom Score and SHIM = Sexual Health Inventory for Men.

	Baseline		3 months		12 months		1 to 2 years	
SHIM Total								
0 to 10	13	20.0%	5	15.6%	13	28.9%	4	19.0%
11 to 20	19	29.2%	12	37.5%	12	26.1%	6	28.6%
> 20	33	50.8%	15	46.9%	20	43.5%	11	52.4%
missing	8		41		28		52	
IPSS Total								
0 to 10	41	63.1%	15	51.7%	34	76.6%	15	71.4%
11 to 20	20	30.8%	9	31.0%	8	17.8%	5	23.8%
>20	4	6.1%	5	17.2%	3	6.7%	1	4.8%
missing	8		44		28		52	
IPSS QOL								
Pleased	29	43.9%	16	55.2%	26	60.5%	11	64.8%
Mostly Satisfied/Mixed	34	51.5%	12	41.3%	14	32.5%	5	29.2%
Dissatisfied	3	4.6%	1	3.5%	3	7.0%	1	6.0%
Missing	7		44		30		56	

**Table 4b:** Comparison of Mean (SD) SHIM and IPSS Scores at Baseline, 12 months Post-op and 24 Months Post-op for Men Undergoing HIFU focal ablation

Quality of Life outcomes	
Mean SHIM Score (SD)	
Baseline	17.0 (7.5)
3 to 12 months Post-op	14.8 (8.3) (p = 0.13)
12 to 24 months Post-op	17.7 (7.9) (p = 0.75)
SHIM Q2 Score (SD)	
Baseline	3.8 (1.6)
3 to 12 months Post-op	3.3 (1.4) (p = 0.13)
12 to 24 months Post-op	3.9 (1.7) (p = 0.57)
Mean IPSS Score (SD)	
Baseline	8.1 (6.0)
3 to 12 months Post-op	7.2 (5.5) (p = 0.42)
12 to 24 months Post-op	7.7 (5.8) (p = 0.81)
Mean IPSS QOL Score (SD)	
Baseline	1.7 (1.3)
3 to 12 months Post-op	1.3 (1.0) (p = 0.06)
12 to 24 months Post-op	1.3 (1.1) (p = 0.26)
