# Supplementary material for: Evaluation of post-ablation mpMRI as a predictor of residual prostate cancer after focal high intensity focused ultrasound (HIFU) ablation
Source: Urol Oncol. Author manuscript; Available in PMC 2023 Mar 29. (PMC10058305; doi:10.1016/j.urolonc.2022.07.017)
Supplement: Supplementary Material [file NIHMS1877509-supplement-Supplementary_Material.docx]

Supplementary Table 1. Characteristics of mpMRI scans utilized in our study

| Scan type | Scan plane | TR (s) | TE (ms) | Field of view (cm) | Matrix | Slice thickness (mm) |
| --- | --- | --- | --- | --- | --- | --- |
| T2 | Axial | 3.7 | 113 | 20 | 320 x 224 | 3 |
| Diffusion* | Axial | 4.5 | 54 | 24 x 12 | 160 x 80 | 4.2 |
| Dynamic post- contrast‡ | Axial | 4 | 1.6 | 34 | 256 x 256 | 2.6 |

*FOCUS, GE Healthcare, Waukesha, WI. b values of 0, 50 (NEX = 3), 800 (NEX = 10), 1400 (NEX = 16) sec/mm2).

‡ T1 GRE fat saturated. Temporal resolution 7 s, 22 phases.
